# The effects of precipitation, river discharge, land use and coastal circulation on water quality in coastal Maine

**DOI:** 10.1098/rsos.140429

**Published:** 2015-07-29

**Authors:** Charles E. Tilburg, Linda M. Jordan, Amy E. Carlson, Stephan I. Zeeman, Philip O. Yund

**Affiliations:** Department of Marine Sciences, University of New England, Biddeford, ME, USA

**Keywords:** *Escherichia coli*, coastal water quality, multiple linear regression models

## Abstract

Faecal pollution in stormwater, wastewater and direct run-off can carry zoonotic pathogens to streams, rivers and the ocean, reduce water quality, and affect both recreational and commercial fishing areas of the coastal ocean. Typically, the closure of beaches and commercial fishing areas is governed by the testing for the presence of faecal bacteria, which requires an 18–24 h period for sample incubation. As water quality can change during this testing period, the need for accurate and timely predictions of coastal water quality has become acute. In this study, we: (i) examine the relationship between water quality, precipitation and river discharge at several locations within the Gulf of Maine, and (ii) use multiple linear regression models based on readily obtainable hydrometeorological measurements to predict water quality events at five coastal locations. Analysis of a 12 year dataset revealed that high river discharge and/or precipitation events can lead to reduced water quality; however, the use of only these two parameters to predict water quality can result in a number of errors. Analysis of a higher frequency, 2 year study using multiple linear regression models revealed that precipitation, salinity, river discharge, winds, seasonality and coastal circulation correlate with variations in water quality. Although there has been extensive development of regression models for freshwater, this is one of the first attempts to create a mechanistic model to predict water quality in coastal marine waters. Model performance is similar to that of efforts in other regions, which have incorporated models into water resource managers' decisions, indicating that the use of a mechanistic model in coastal Maine is feasible.

## 1. Introduction

Faecal pollution from humans, pets and domesticated animals in stormwater, wastewater and direct run-off can carry zoonotic pathogens to streams, rivers and the ocean [1,2]. Shellfish beds, fish hatcheries and beach systems can be affected by faecal pollution within rivers and the buoyant plumes emanating from those and other sources of freshwater to the coastal ocean [3–5]. The detection of the zoonotic protozoan parasites, *Giardia* and *Cryptosporidium* [6,7], and pathogenic bacteria, *Campylobacter*, *Vibrio* and *Salmonella* [8], in water and bivalve samples from estuarine and coastal locations indicates that faecal pollution and the associated pathogens are emerging coastal public health problems.

A number of studies have confirmed the link between precipitation and increased microbial pollution, which can result in reduced water quality in rivers, estuaries and coastal environments (e.g. [9–12]). *Giardia* and *Cryptosporidium* contamination are typically associated with heavy precipitation events and the concomitant freshwater run-off. One study found an association between extreme rainfall events and monthly reports of outbreaks of waterborne disease [13]. Others have reported the detection of *Cryptosporidium* spp. in mussels [7] and oysters [6] after heavy rainfalls.

Land use has a direct effect on the relationship between precipitation, run-off and water quality [14,15]. Land use changes can alter regional weather patterns and vegetation in adjacent natural areas, primarily through agriculture and urbanization [16]. Impermeable surfaces of urbanized areas (e.g. [17]) increase run-off and the transport of pollutants into streams, rivers and the ocean. Changes in land use can result in greater erosion, more run-off and increased turbidity in rivers (e.g. [18]). The strong relationship between land use and *Giardia* and *Cryptosporidium* contamination of water may be related to agricultural land use [19].

In addition to precipitation and river discharge, other physical mechanisms may be responsible for the concentration or dispersal of pathogens in estuaries and the coastal ocean [20,21]. The coastal ocean is affected by tides, wind-driven transport and river plumes (e.g. [22,23]), which can transport pollutants from their sources to new locations, resulting in reduced water quality far from the source of the pollution (e.g. [24]). A number of studies have investigated the transport of larvae [25–27] and sediment [28,29] in the coastal waters of Maine. Although river plumes are important pathways for the transport of faecal pollution and the accompanying pathogens by river plumes (e.g. [30–32]), the examination of the specific mechanisms responsible for this transport has just begun (e.g. [5]).

The threats to human health from pathogens have increased [10,33]. Reductions in water quality after high precipitation events and the subsequent increases in river discharge have led local authorities to close beaches and shellfish beds after large events in coastal Maine (e.g. [34,35]) and other locations throughout North America [36–38]. The inability of local authorities to accurately predict these events or to immediately assess the water quality exacerbates the losses to fishing and tourist economies in these regions [36–38].

The need for accurate and timely predictions of water quality in the United States has become acute [11]. However, the testing for the presence of faecal bacteria is labour intensive and generally requires an 18–24 h period for sample incubation. Water quality may change during that time period [39], which could lead to inaccurate water quality advisories resulting in exposure to pathogens in the water or unnecessary closures.

The reliance on a single parameter for the prediction of water quality can be problematic, requiring more complex, predictive models. Sampson *et al*. [40] found no relationship between rainfall events and faecal bacteria at Lake Superior recreational beaches. Studies have examined various inputs on water quality using partial least-squares regression models [41], artificial neural networks [4] and multiple linear regression models [3,42]. Although there has been extensive development of predictive models for freshwater (e.g. [3,42,43]), applications to marine waters have been limited (e.g. [4,41,44]).

In this study, we continue the examination of the relationship between water quality and hydrometeorological processes such as wind-, buoyancy- and tidally driven transport in the marine waters. Our regions of study are Saco Bay and the region within and adjacent to the Kennebec and Androscoggin Rivers. Both sites are located in the Gulf of Maine. The central objectives of this study were: (i) to perform an historical examination of water quality at stations within shellfish growing areas in Saco Bay and the mouth of the Kennebec and Androscoggin river system during 1998–2010 (hereafter referred to as study I), (ii) to examine the effect of land use, river discharge, precipitation at various locations within the watershed and coastal circulation patterns on faecal bacterial contamination within Saco Bay during 2010–2012 (here after referred to as study II), and (iii) to construct and test the first regression model that could be used to predict water quality at five stations in the Saco River and Saco Bay during study II.

To achieve our objectives, we use remotely sensed and *in situ* data of the Gulf of Maine watersheds, rivers, estuaries and coastal ocean. Analysis of a 12 year dataset revealed that high river discharge and/or precipitation events can lead to reduced water quality at multiple locations along the coast; however, the use of only these two parameters resulted in a number of prediction errors. Analysis of the higher frequency, 2 year study revealed that precipitation, salinity, river discharge, winds, seasonality and coastal circulation have significant effects on water quality. We show that a multiple regression model based on readily obtainable measurements can be used in the prediction of water quality events at five locations within Saco Bay and the Saco River mouth.

## 2. Material and methods

### 2.1 Study area

**Figure 1.**
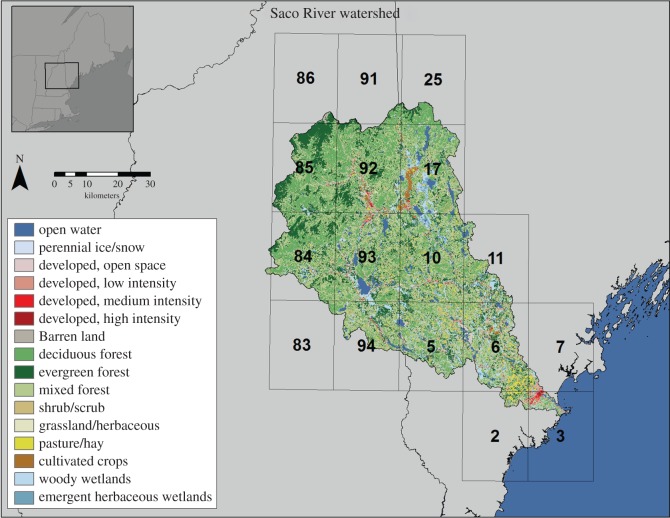
Land use characteristics of the Saco River watershed. The numbers indicate locations of TRMM measurements.

Our study sites encompass the watersheds, mouths and adjacent coastal ocean of (i) the Saco River and (ii) the combined Kennebec and Androscoggin river system. The mean discharge rates of the Saco, Androscoggin and Kennebec rivers are 70, 175 and 258 m^3^ s^−1^, respectively. The watersheds of the three rivers occupy approximately 4410, 8975 and 15 270 km^2^ [45]. All three watersheds are characterized by little development; however, the Saco River watershed (figure 1) is more developed than the others. Furthermore, 9.1% of the land in the Saco River watershed is developed versus 1.6% and 2.0% for the Kennebec and Androscoggin watersheds, respectively [46]. All three rivers empty into the southwestern portion of the Gulf of Maine, a marginal sea in the northwest Atlantic Ocean. The Saco River enters the relatively open Saco Bay [28] through a narrow mouth (approx. 250 m) flanked by two jetties, resulting in a discrete point of entry, generating a plume that is 1–2 m deep, is highly mobile and extends 5–12 km into the Gulf of Maine [47]. The Saco plume is the primary source of freshwater for that bay [47], although the much smaller Scarborough River also empties into the northern end of Saco Bay. The Kennebec and Androscoggin Rivers combine in Merrymeeting Bay before discharging into the Gulf of Maine. Their plume is substantially larger than that of the Saco River and can extend more than 30 km into the Gulf of Maine [48]. The water conditions at the river mouths and within the adjacent coastal ocean of both river systems are governed by wind-driven transport, tidal currents, small-scale buoyancy forcing from the plumes emanating from the river mouths and large-scale buoyancy forcing from the Gulf of Maine coastal current system [22,23]. Both regions are home to a number of shellfish growing areas whose access by fishermen is determined by the State of Maine Department of Marine Resources (DMR) based on predicted and/or measured pollution levels. Saco Bay also contains a number of beaches that attract tourists from Canada and the northeast USA. The major sources of faecal pollution to Saco River are four wastewater treatment plants (WWTPs) and a storm water outfall, as well as licenced overboard discharges and non-point sources in the watershed [34]. The major sources of faecal pollution to the Kennebec and Androscoggin river system are 10 WWTPs, as well as a number of private in-ground septic systems, composting toilets and licenced overboard discharges [35].

### 2.2 Data

**Figure 2.**
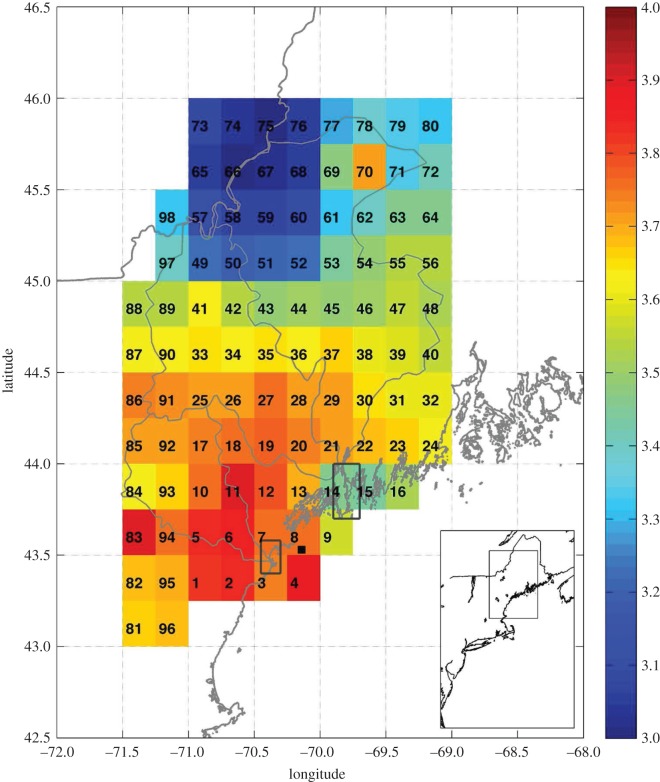
Mean daily precipitation (mm) from 5 January 1998 to 5 January 2013. The numbers indicate locations of TRMM measurements. The black square located in TRMM square 8 indicates the location of Environmental Buoy no. 44007. The two rectangles indicate the regions shown in figures 3, 5 and 7. The grey lines indicate the boundaries of the Saco, Androscoggin and Kennebec River watersheds (from southwest to northeast). Inset shows location of the study regions.

Hydrometeorological data were obtained using a combination of *in situ* and remotely sensed methods. Daily remotely sensed precipitation values were obtained from the Tropical Rainfall Measuring Mission (TRMM) and Other Rainfall Estimate (3B42 version 2 derived) Online Visualization and Analysis System (TOVAS) for the time period 1998–2012. The precipitation values for both studies I and II were collected at a resolution of 0.25°×0.25° throughout the watersheds (see figure 2 for spatial coverage and labelling convention for this study). Throughout this paper, the locations of the precipitation measurements are referenced by the number of the TRMM square (e.g. TRMM square 53). Saco River discharge data were obtained from the United States Geological Survey (USGS) gauge located at Cornish, ME. Kennebec and Androscoggin River discharge data were obtained from USGS gauges located at North Sydney, ME and Auburn, ME, respectively. As the water quality stations in the Kennebec River and Androscoggin River system were located downstream of Merrymeeting Bay where the two rivers joined, all calculations for discharge use the combined discharges and all examination of watershed processes treat the two watersheds as one (hereafter the Kennebec River and Androscoggin River system is referred to as KA). Salinity in study II was measured using a handheld refractometer and quantified at the time of water quality sampling. Mean wind speed and direction for study II were calculated from meteorological data obtained from NOAA Environmental Buoy no. 44007 located in the Gulf of Maine between the two study sites (small black square located in TRMM square 8 in figure 2).

Land use was classified using imagery from Landsat 1 to present. All Landsat images were projected into the UTM zone 19N coordinate system, which spans the entire study area. Atmospheric correction was performed on all spectral images using the COS(T) dark object subtraction method [49]. For each image, ground location comparison was performed visually between the satellite data and a Maine roads layer. Land-cover classes include deciduous forest, evergreen forest, mixed forest, herbaceous wetlands, forested wetland, shrub/scrub, cultivated crops, pasture or hay, low-intensity developed, medium-intensity developed, high-intensity developed and water. These 10 categories were chosen based on prior knowledge of the area's most common and dominant land-cover types, which can be easily identified in both ortho-photography and spectral colour composites and which have the most spectrally separable signatures.

**Figure 3.**
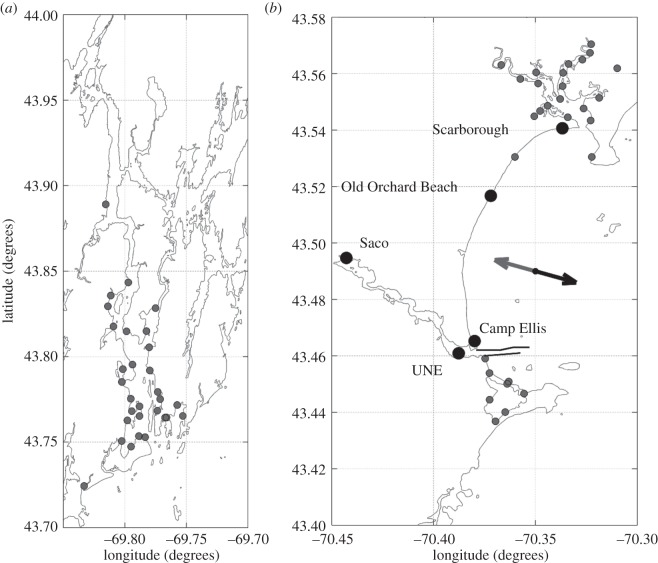
Location of the water quality stations from study I (small grey circles) and study II (large black circles) in the mouths of the Kennebec and Androscoggin Rivers (*a*) and the Saco River (*b*). The black lines in panel (*b*) indicate the locations of the jetties. The grey arrow in panel (*b*) shows the direction of movement of the river plume due to flood tides and easterly/northeasterly winds. The black arrow shows the direction of the river plume due to ebb tides and westerly winds.

Faecal coliforms were sampled by the State of Maine DMR from 1998 to 2010 for stations within the mouths of the KA system (figure 3*a*) and the Saco River (figure 3*b*) at approximately monthly intervals throughout the spring, summer and early autumn. The sampling and processing protocols during this time period in the Saco and the KA systems are outlined in the WM Triennial Review [35] and the WG Triennial Review [34], respectively. All sample stations for study I were chosen from previously monitored stations based on their proximity to the mouths of the rivers and shellfish growing beds.

Sampling for study II occurred between November 2010 and November 2012 and was limited to surface waters near the mouth of the Saco River. This study consisted of much higher temporal resolution sampling (i.e. every 2–3 days) to capture short time-scale features in the region. Samples were collected and tested for both *Escherichia coli* and total coliforms. Three sample stations were located on the beaches along the coast of Saco Bay and two stations were located in the Saco River (large black circles in figure 3*b*). Three replicate water samples were collected at 1 m depth at each station between November 2010 and November 2012 using sterilized 250 ml glass bottles. Sampling was discontinued during the winter when precipitation was mostly frozen. Samples were processed by membrane filtration and then cultured in m-ColiBlue24 media (Hach Company, Loveland, CO, USA). This media was selected because it yields accurate counts for both *E. coli* and total coliforms (but unfortunately not *Enterococcus*) in both fresh and salt water [50]. Because we were sampling across a salinity gradient that spanned the range from fresh to salt water, we felt that it was more important to use a single culture medium that was insensitive to salinity rather than maximize taxonomic coverage. Following a 24 h incubation at 37°C, *E. coli* density was assessed by counting the total number of blue-stained colonies, whereas total coliform density was assessed by summing counts for both red and blue colonies. Concentrations at each station in study II are expressed as the log of the mean values calculated from the three replicates.

### 2.3 Statistical methods and models

The relationships between water quality and the hydrometeorological parameters of the region were examined by: (i) a decision model that linked high river discharge events with reduced water quality events (RWQEs) during study I, (ii) a decision model that linked high precipitation events with RWQEs during study I, (iii) direct correlation calculations of *E. coli* and total coliform concentrations with physical–chemical parameters during study II, and (iv) a multiple linear regression model relating river discharge, salinity, wind direction and magnitude, precipitation and seasonality to *E. coli* and total coliform concentrations during study II.

Both the low temporal resolution of the water quality measurements during study I and the probable complex relationships between water quality and the physical mechanisms of precipitation and discharge prevented a straightforward correlation comparison. Instead, we determined the relationship between periods of elevated concentration of faecal coliforms and either high precipitation or high discharge events using decision models to examine the effects of variable amounts of river discharge or precipitation within the watershed on water quality at stations in study I. The decision models allowed us to test the hypotheses that a RWQE (defined as the time when faecal coliforms within a sample exceeded 14 colonies per 100 ml, which is the maximum allowable value for shellfish growing areas for the state of Maine [51]) occurs within a set period of time after a high discharge event or after a high precipitation event within the watershed. Decision models were created that predicted a RWQE at a water quality station would occur within a designated time period if: (i) river discharge preceded a predetermined value, or (ii) precipitation exceeded a predetermined value at a designated TRMM square. The predetermined values of discharge and precipitation as well as the location of the TRMM square in which the precipitation was measured were systematically varied to achieve the best results for each model. Once the model was created, we then determined: (i) the fraction of observed RWQEs that were predicted by high discharge or high precipitation events, and (ii) the fraction of high discharge or high precipitation events that did not precede a RWQE (i.e. a false alarm or a type I error [52]). A perfect model would predict all RWQEs, resulting in no exposure to faecal coliforms and potential pathogens (or type II errors [52]), and produce no false alarms or unnecessary closures of the region.

To determine the significance of the decision models, we used a simple randomization test, in which we compared the ability of observed precipitation and discharge events to predict RWQEs with distributions created from a large number of synthetic datasets. The synthetic datasets for each station were constructed by: (i) determining the number of observed RWQEs at each station in the actual dataset, (ii) placing the same number of RWQEs at random times throughout the study period, and (iii) calculating the fraction of these randomly placed RWQEs that were preceded by high discharge or high precipitation events and the percentage of times in which a high discharge or precipitation event did not precede a RWQE, or a false alarm. Once a large number (i.e. 1000) of datasets were created, frequency distributions of the fraction of RWQEs that followed a high discharge or precipitation event and the fraction of false alarms were created. Comparison of the performance of the models using actual discharge and precipitation data to the frequency distributions of the synthetic datasets provided an estimate of the significance of the models' ability to predict RWQEs. For example, a model whose percentage of predicted RWQEs was greater than 95% of the synthetic datasets and whose number of false alarms was less than 95% of the synthetic datasets was deemed to outperform random chance at a significance of 95% (e.g. [53,54]). The models were calibrated for each station by selecting: (i) the precipitation amount at the TRMM square, or (ii) the discharge value that resulted in the highest significance for that station.

Pearson moment correlations between the water quality parameters (*E. coli* and total coliforms) and the hydrometeorological parameters (river discharge, salinity, wind direction and magnitude, and precipitation measured at different TRMM squares) were calculated for all stations in study II. In addition, correlations were calculated between the water quality parameters and a simple cosine function to examine seasonality of the water quality. The cosine function was expressed as

2.1 $$T(t)=\cos\left(\frac{2\pi t}{A}\right),$$

where *t* is day of year, and *A* is 365 days.

When examining the multiple correlations between the hydrometeorological and water quality time series, we used the modified Bonferroni method described by Rice [55] to maintain a 95% significance level across the entire table of tests, rather than at the level of the individual test.

As the stations surrounding the Saco River in study II were sampled with much higher temporal resolution, we were able to construct multiple linear regression models [42,56] that predict *E. coli* and total coliform concentrations at all five stations in the region using the hydrometeorological parameters and seasonality. The equation representing the model for each station is shown below:

2.2 $$M_{c}(t)=C_{o}+\sum_{j=1}^{n}C_{j}X_{j}(t-\zeta_{j})+e(t),$$

where *M*_c_(*t*) is the estimated *E. coli* or total coliform concentration at the station, *n*=5 for the Saco station and 6 for all other stations, *X*_1_(*t*) is the daily discharge (m^3^ s^−1^) from the Saco River, *X*_2_(*t*) is the salinity at the station, *X*_3_(*t*) is the wind velocity (m s^−1^) measured at EB 44007, *X*_4_(*t*) is the daily precipitation measured at a TRMM square (mm), *X*_5_(*t*) is the seasonality described in equation (2.1), *X*_6_(*t*) is the observed *E. coli* or total coliform concentration at the Saco station, *ζ*_1_, *ζ*_2_, *ζ*_3_, *ζ*_4_, *ζ*_5_ and *ζ*_6_ are the time lags (days) associated with each parameter, *C*_*o*_ is a constant, *C*_1_, *C*_2_, *C*_3_, *C*_4_, *C*_5_ and *C*_6_ are the regression coefficients that were determined using least-squares fit methods, and *e*(*t*) is an error term.

## 3. Results

### 3.1 Hydrometeorological data

During both studies, precipitation varied both spatially and temporally throughout the region. The average daily precipitation (figure 2) ranged from a minimum of 3.02 mm at TRMM square 75 to a maximum of 3.92 mm at TRMM square 83. The Saco River watershed (southwesternmost watershed) received more rainfall than the other watersheds. River discharge and the total precipitation over the entire watersheds are linked. Monthly averages of discharge and total precipitation were correlated in both the Saco River watershed (*r*=0.38, *p*<0.001) and the Kennebec River and Androscoggin River watersheds (*r*=0.42, *p*<0.001).

**Figure 4.**
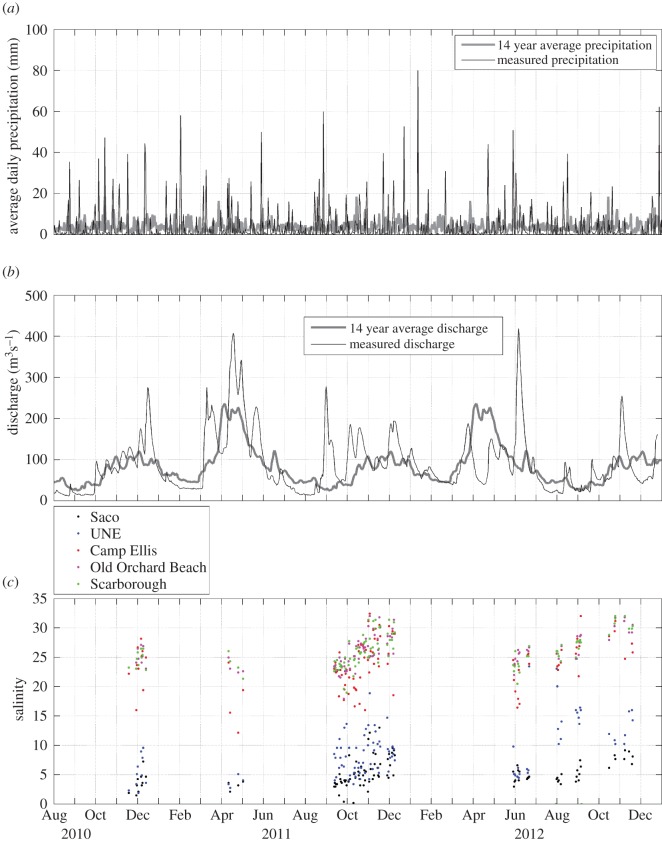
(*a*) Average daily precipitation (mm) over the Saco River watershed obtained from TRMM squares, (*b*) discharge (m^3^ s^−1^) of Saco River measured at USGS gauging station in Cornish, ME, (*c*) salinity at stations within Saco River and Saco Bay, during study II.

Examination of the precipitation throughout the Saco River watershed during study II (black line in figure 4*a*) reveals strong daily variation. The 2 years of study II were wetter than the 14 year average from 1998 to 2012 (grey line in figure 4*a*). Discharge in the Saco River (black line in figure 4*b*) shows a peak in the late spring/early summer of both years that corresponds to the spring freshet as well as a number of other peaks that typically follow precipitation events. Consistent with the observed precipitation, river discharge during study II was typically greater than the 14 year average (grey line in figure 4*b*). Examination of the surface salinity (figure 4*c*) in the Saco River region reveals that stations in the river (Saco and University of New England (UNE) stations) were consistently fresher than those in the coastal ocean (Old Orchard Beach and Scarborough stations) and at the mouth (Camp Ellis station; figure 3*b*). Salinities at all stations were significantly correlated with each other (table 1) but not with discharge or precipitation. Salinity correlations were substantially higher among the two coastal ocean stations and Camp Ellis than between those stations and the two river stations (table 1).

**Table 1.** Correlations of salinities between stations.

	Saco	UNE	Camp Ellis	Old Orchard Beach	Scarborough
Saco	1.00	0.40^a^	0.63^a^	0.69^a^	0.68^a^
UNE		1.00	0.47^a^	0.42^a^	0.45^a^
Camp Ellis			1.00	0.80^a^	0.83^a^
Old Orchard Beach				1.00	0.94^a^
Scarborough					1.00

^a^Indicates the correlation is significant at 99% using the sequential Bonferroni method [55].

### 3.2 Water quality data

**Figure 5.**
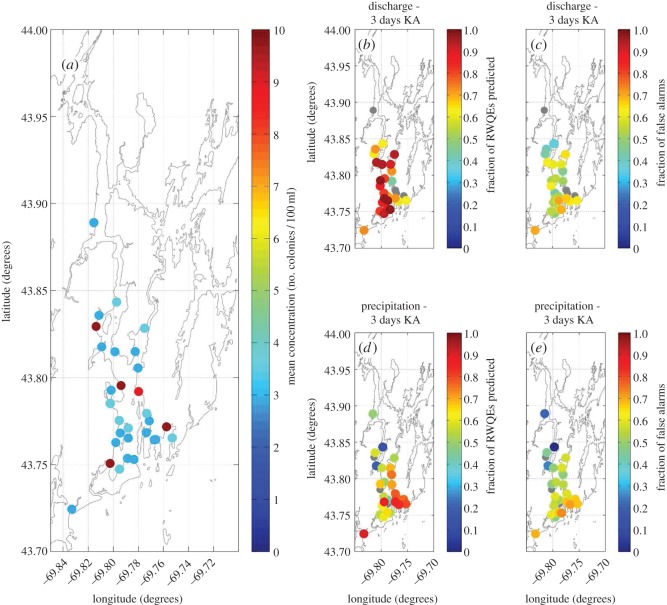
Mean concentrations of faecal coliforms (*a*) measured at stations in the mouths of the Kennebec and Androscoggin Rivers. (*b*) Fraction of RWQEs preceded by a large discharge event in the previous 3 days. (*c*) Fraction of times that a large discharge event did not result in a RWQE in the next 3 days, i.e. a ‘false alarm’. (*d*) Fraction of RWQEs preceded by a large precipitation event in the previous 3 days. (*e*) Fraction of times that a large precipitation event did not result in a RWQE in the next 3 days, i.e. a ‘false alarm’ (*e*). Grey circles indicate stations whose percentage of RWQEs or percentage of false alarms was not significant at 95%.

**Figure 6.**
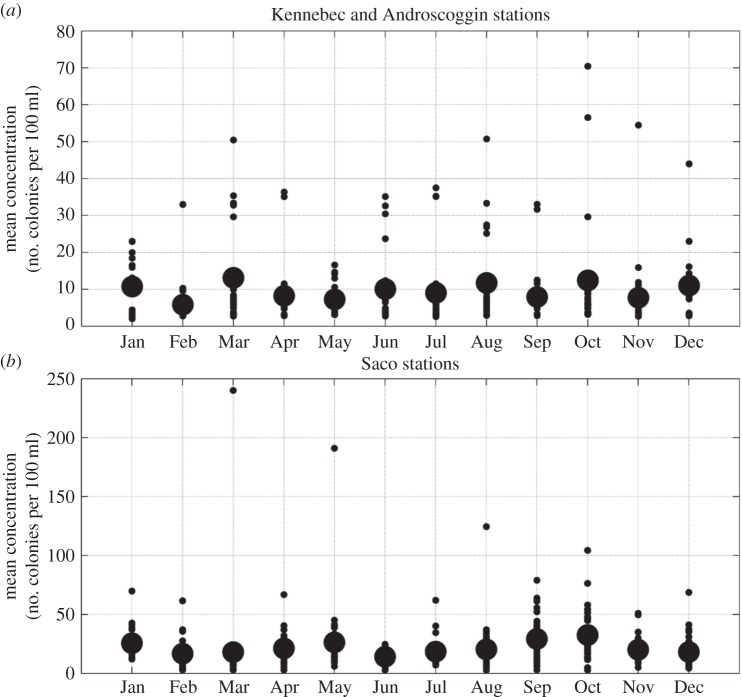
Monthly averages of each station in Kennebec and Androscoggin River watersheds (*a*) and in Saco River watershed (*b*). Small filled circles represent monthly averages of individual stations. Large filled circles represent monthly average of all stations.

During study I, water quality in the KA system mouth was characterized by large variations in space and time. Mean concentrations of faecal coliforms (figure 5*a*) were less than 4 colonies per 100 ml for most locations; however, some locations near the coast and far inland were characterized by higher concentrations. Examination of the average concentrations of all stations shows a weak seasonal signal with slight peaks in March and October (figure 6*a*).

**Figure 7.**
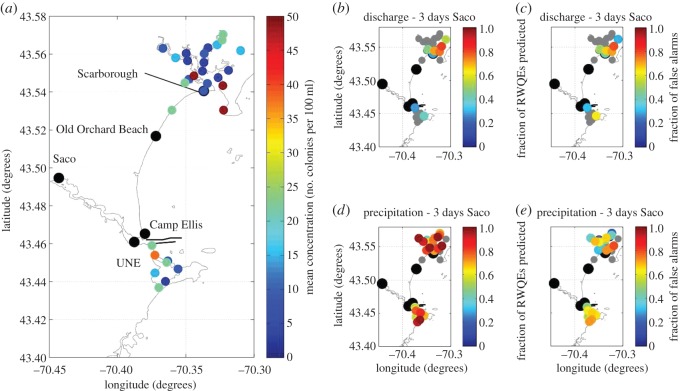
Mean concentrations of faecal coliforms (*a*) measured at stations in the mouth of the Saco River. (*b*) Fraction of RWQEs preceded by a large discharge event in the previous 3 days. (*c*) Fraction of times that a large discharge event did not result in a RWQE in the next 3 days, i.e. a ‘false alarm’. (*d*) Fraction of RWQEs preceded by a large precipitation event in the previous 3 days. (*e*) Fraction of times that a large precipitation event did not result in a RWQE in the next 3 days, i.e. a ‘false alarm’. Note that large black circles indicate location of stations measured during the years 2010–2012. Grey circles indicate stations whose percentage of RWQEs or percentage of false alarms was not significant at 95%.

Examination of the mean concentration of faecal coliforms in Saco Bay during study I reveals higher values than those found within the Kennebec and Androscoggin Rivers (figure 6*b*), with a number of stations that exceed 50 colonies per 100 ml (figure 7*a*). In contrast to the KA system, the highest concentrations were found along the coast; however, there was no sampling upstream in the Saco River (where four WWTPs discharge) during study I. Examination of average concentration of the region also found a seasonal signal, with peaks in May and October (figure 6*b*).

The complex relationship between the transport of faecal pollution and physical mechanisms combined with the low frequency of measurements in study I resulted in no significant correlation between faecal coliforms and discharge or precipitation in either region. Consequently, we employed a decision model to determine if either high values of river discharge or high values of precipitation within the watersheds predicted RWQEs at the different stations at the river mouths. The decision model was first calibrated to determine the optimal discharge (or precipitation amount) that triggered a RWQE and the optimal response period (i.e. the time during which a RWQE would occur after a high discharge of precipitation event). The values of discharge and precipitation that preceded RWQEs varied by station (e.g. high values of river discharge were 75–500 m^3^ s^−1^, while high values of precipitation were 8–20 mm), but a response period of 3 days was optimal for all stations in both regions.

Examination of the fraction of observed RWQEs in the Kennebec and Androscoggin Rivers that were correctly predicted by the model (figure 5*b*) and the fraction of false alarms (figure 5*c*) reveals that large discharge events occurred within 3 days of RWQEs (at 95% significance) at 25 out of 30 locations. The decision model using precipitation as input showed that large precipitation events occurred within 3 days of RWQEs (at 95% significance) at 28 out of 30 locations (figure 5*d*,*e*).

The decision model was not as effective in the Saco River watershed. The decision model using discharge as input showed that large discharge events occur within 3 days of RWQEs (at 95% significance) at eight out of 31 locations (figure 7*b*,*c*). The decision model using precipitation as an input showed that large precipitation events occurred within 3 days of RWQEs (at 95% significance) at 23 out of 31 locations (figure 7*d*,*e*).

**Figure 8.**
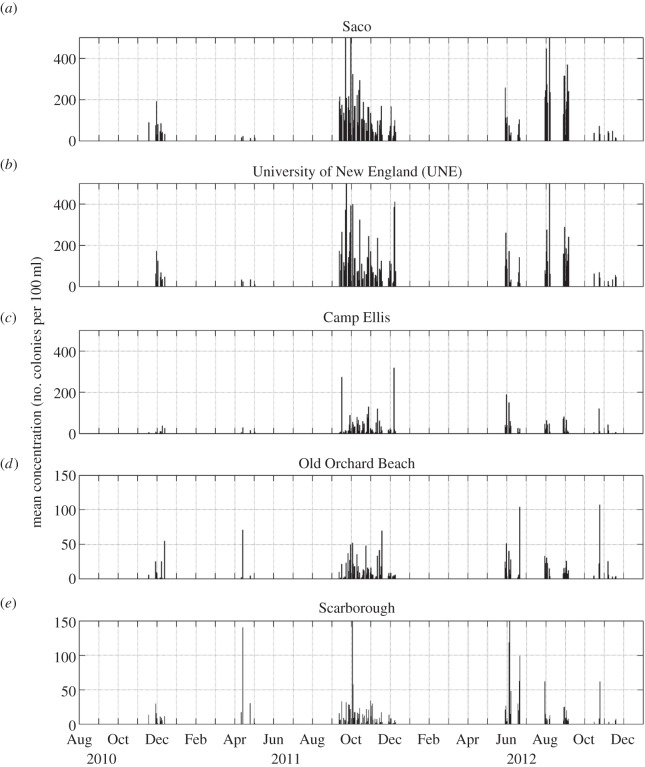
(*a*–*e*) Daily concentrations of *E. coli* from 9 November 2010 to 30 November 2012 at the five stations in study II.

**Figure 9.**
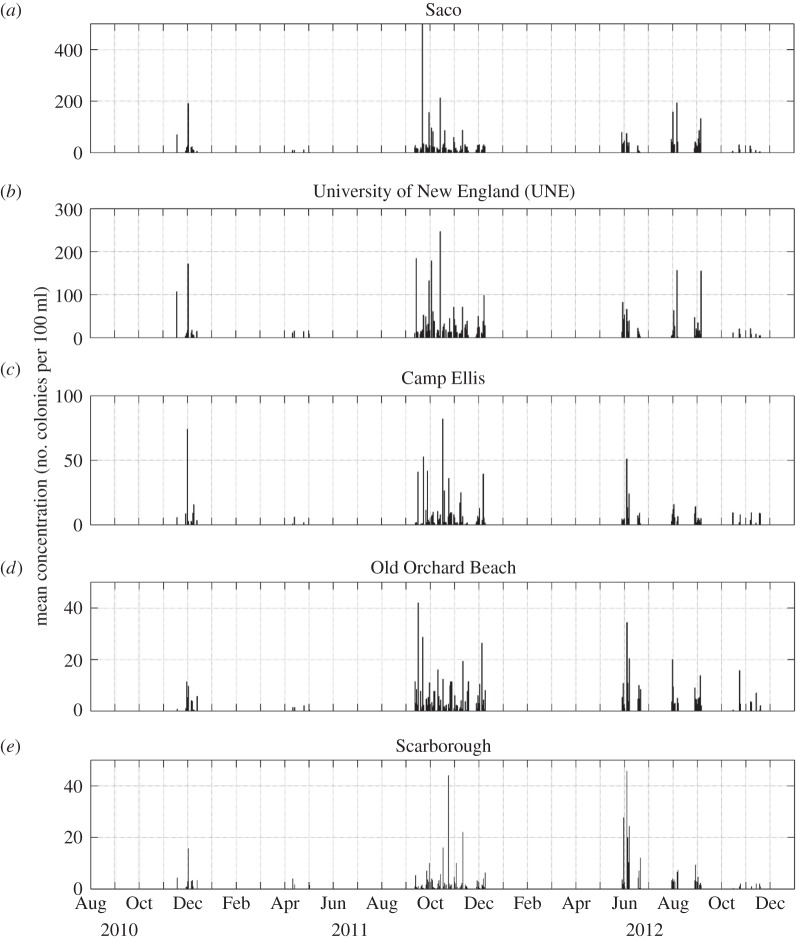
(*a*–*e*) Daily total coliform concentrations from 9 November 2010 to 30 November 2012 at the five stations in study II.

Examination of the *E. coli* concentrations (figure 8) and total coliform concentrations (figure 9) collected at the five locations within Saco Bay from 2010 to 2012 during study II reveals strong temporal and spatial variation. The two stations within the river (Saco and UNE stations) were characterized by high concentrations of both *E. coli* and total coliforms. The station at Camp Ellis, which is near the mouth of the Saco River but separated from the river by a jetty that is under water only during spring high tides (figure 3*b*), was characterized by lower concentrations. The other two stations along the beach (Old Orchard Beach station) and further north of the Saco River (Scarborough station) were characterized by even lower concentrations. As in study I, all five stations had a seasonal signal in both *E. coli* and total coliforms. At all locations, the largest values were found during late summer/early autumn; however, the coastal and Camp Ellis stations had an additional peak in early summer. An examination of the correlations of *E. coli* concentrations between stations (table 2) reveals that the river stations are significantly correlated with concentrations at adjacent stations and the two coastal stations are correlated with each other, but those stations within and near the river (Saco, UNE and Camp Ellis stations) were not correlated with those along the coast (Old Orchard Beach and Scarborough stations). Total coliform concentrations (table 3) show less spatial correlation. While total coliform concentrations at the Saco station were significantly correlated with concentrations at the UNE and Old Orchard Beach stations and the two coastal stations were correlated, the total coliform concentrations at the Camp Ellis station were not correlated with any other station.

**Table 2.** Correlations of log *E. coli* concentrations between stations.

	Saco	UNE	Camp Ellis	Old Orchard Beach	Scarborough
Saco	1.00	0.67^a^	0.024	0.022	0.014
UNE		1.00	0.31^a^	0.05	0.10
Camp Ellis			1.00	0.18	0.11
Old Orchard Beach				1.00	0.40^a^
Scarborough					1.00

^a^Indicates the correlation is significant at 95% using the sequential Bonferroni method [55].

**Table 3.** Correlations of log total coliform concentrations between stations.

	Saco	UNE	Camp Ellis	Old Orchard Beach	Scarborough
Saco	1.00	0.41^a^	−0.064	0.30^a^	0.082
UNE		1.00	−0.034	0.026	0.15
Camp Ellis			1.00	0.24	0.25
Old Orchard Beach				1.00	0.31^a^
Scarborough					1.00

^a^Indicates the correlation is significant at 95% using the sequential Bonferroni method [55].

Meteorological and oceanographic processes were correlated with *E. coli* concentrations (table 4). River discharge was negatively correlated with *E. coli* concentrations at stations within the river (*r*=−0.52 and *r*=−0.42 for Saco and UNE stations, respectively) but not at stations out of the river. Salinity was negatively correlated with *E. coli* concentrations at the Saco station but not at any other stations. Winds were correlated with *E. coli* concentrations along the coast and Camp Ellis but not with those inside the river. The highest correlations of *E. coli* concentrations occurred with winds from the northeast (i.e. 30–50°N) at a lag of 1–2 days. Precipitation was correlated with *E. coli* concentrations at the Camp Ellis and coastal stations. The locations of precipitation with the highest correlation varied from far up the watershed (*E. coli* concentrations at the Camp Ellis station showed the highest correlation with TRMM square 93) to the immediate region (concentrations at the Scarborough station exhibited the highest correlation with TRMM square 3). In agreement with the seasonal signal found in the monthly averages of study I, *E. coli* concentrations at all five stations were significantly correlated with a simple cosine curve that simulates seasonality (equation (2.1)), although the lag time (which determined the timing of the peak values of the cosine curve) varied by station (table 4) and ranged from year day 193 (11 July) to year day 269 (25 September). Construction of a multiple linear regression model that includes the hydrometeorological time series (equation (2.2)) to predict *E. coli* concentrations (table 4) at the five stations resulted in correlations between the model and *E. coli* concentrations that range from 0.47 to 0.76. Including *E. coli* concentrations at the Saco station in the regression model improved the correlation at the UNE station, raising the correlation from 0.48 to 0.56, and had smaller effects on the Camp Ellis and coastal stations. *Escherichia coli* concentrations lagged the hydrometeorological time series by 0–3 days.

**Table 4.** Correlations between hydrometeorological variables and log *E. coli* concentrations at all stations.

	discharge	salinity	winds	precipitation	season	total	total^b^
Saco	−0.52^a^ (3 days)	−0.28^a^	0.20 (140°N, 4 days)	0.24 (0 days, TRMM 3)	0.69^a^ (269 days)	0.76^a^	0.76^a^
UNE	−0.42^a^ (2 days)	0.17	0.13 (160°N, 2 day)	0.15 (0 days, TRMM 84)	0.28^a^ (267 days)	0.48^a^	0.56^a^
Camp Ellis	0.13 (0 days)	−0.20	0.42^a^ (30°N, 1 day)	0.29^a^ (2 days, TRMM 93)	0.23 (226 days)	0.55^a^	0.56^a^
Old Orchard Beach	−0.20 (6 days)	−0.066	0.30^a^ (30°N, 2 days)	0.29^a^ (1 day, TRMM 10)	0.19 (229 days)	0.47^a^	0.47^a^
Scarborough	0.20 (0 days)	−0.22	0.52^a^ (50°N, 2 days)	0.33^a^ (3 days, TRMM 3)	0.41^a^ (193 days)	0.62^a^	0.63^a^

^a^Indicates the correlation is significant at 95% using the sequential Bonferroni method [55].

^b^Includes log *E. coli* concentrations from Saco station.

Meteorological and oceanographic processes were also correlated with total coliforms (table 5), but to a lesser degree. River discharge was negatively correlated with total coliform concentrations at only the Saco station. Salinity was not significantly correlated with total coliform concentrations at any station. Winds were correlated with total coliform concentrations at all locations but Camp Ellis. The highest correlation occurred with winds from the east–southeast (i.e. 110–120°N) at a lag of 4–5 days. Precipitation was correlated with total coliform concentrations at the Saco and Scarborough stations. In contrast to *E. coli*, total coliforms were correlated with precipitation only within the immediate region (i.e. TRMM square 3). Total coliform concentrations at the Saco and Scarborough stations were significantly correlated with a simple cosine curve that simulates seasonality (equation (2.1)). Construction of a multiple linear regression model (equation (2.2)) for total coliforms (table 5) at the five stations resulted in correlations that ranged from 0.38 to 0.65. Including total coliform concentrations at the Saco station (the approximate point of origin for coliforms release from WWTPs) in the regression model improved the correlation at the UNE station, raising the correlation from 0.39 to 0.56, and had smaller effects on the Camp Ellis and coastal stations. Total coliform concentrations lag the hydrometeorological time series by 0–5 days.

**Table 5.** Correlations between hydrometeorological variables and log total coliform concentrations at all stations.

	discharge	salinity	winds	precipitation	season	total	total^b^
Saco	−0.44^a^ (6 days)	−0.14	0.33^a^ (120°N, 4 days)	0.34^a^ (0 days, TRMM 3)	0.35^a^ (260 days)	0.65^a^	0.65^a^
UNE	−0.10 (6 days)	−0.17	0.27^a^ (110°N, 4 days)	0.26 (1 day, TRMM 17)	0.049 (242 days)	0.39^a^	0.56^a^
Camp Ellis	0.080 (0 days)	−0.12	0.26 (50°N, 1 day)	0.22 (6 days, TRMM 8)	0.071 (238 days)	0.38^a^	0.38^a^
Old Orchard Beach	−0.087 (3 days)	0.021	0.28^a^ (120°N, 5 days)	0.17 (5 days, TRMM 11)	0.25 (238 days)	0.38^a^	0.41^a^
Scarborough	0.23 (0 days)	−0.089	0.44^a^ (110°N, 5 days)	0.38^a^ (4 days, TRMM 3)	0.34^a^ (171 days)	0.57^a^	0.60^a^

^a^Indicates the correlation is significant at 95% using the sequential Bonferroni method [55].

^b^Includes log total coliform concentrations from Saco station.

Examination of both *E. coli* and total coliform concentrations revealed a number of similarities. Both concentrations were lower at stations farther from the Saco River. Both were affected by river discharge, winds, precipitation and seasonality. The Camp Ellis and coastal stations experienced peaks in both *E. coli* and total coliform concentrations during the summer, whereas those in the river peaked in the autumn. Both *E. coli* and total coliform concentrations in some river stations were negatively correlated with discharge but the coastal stations were relatively unaffected by discharge. Finally, salinity had a weak effect on *E. coli* concentrations, but no effect on total coliforms. In contrast to other studies (e.g. [3,4,42]), we found no relationship between solar insolation, measured wave height, or sea level with water quality in the region.

## 4. Discussion

Water quality in coastal Maine is governed by a number of physical and biological mechanisms that can be measured or predicted with varying degrees of difficulty. While water quality is affected by biological factors that are not easily (or quickly) measured, a significant amount of the variability in water quality can be explained by easily measured (some remotely) physical processes.

Examination of those processes that determine the transport and circulation in the coastal regions adjacent to the mouths of the rivers provides a better understanding of the mechanisms responsible for variation in water quality in the rivers and coastal ocean. The long time-scale (more than 1 day) circulation patterns within Saco Bay (and the coastal region adjacent to the Androscoggin and Kennebec Rivers) are dominated by river discharge and wind-driven transport (e.g. [22,23,47]). Examination of salinities in study II shows that stations within the Saco River are consistently fresher than those along the coast of Saco Bay. The correlations between salinities at the two coastal stations and those in the river suggest that the plume emanating from the mouth of the Saco River affects the salinity structure of the entire Saco Bay. Short time-scale (less than 1 day) circulation patterns within Saco Bay are dominated by tides [47,57]. The lack of correlation between salinity (which was measured at various times during the day and at different tidal phases) and either discharge or precipitation (which were collected and calculated as daily averages) is consistent with a region whose short-term circulation is governed by tidal transport.

Examination of average values of faecal coliforms during study I (figure 5*a*) reveals that the stations within the Kennebec and Androscoggin rivers were consistently lower than those in the Saco River mouth (figure 7*a*). This difference is consistent with the greater development and greater population density within the Saco River watershed (e.g. [14,15]).

The lack of correlation between the measured faecal coliforms and either discharge or precipitation in study I is most likely owing to a combination of the low temporal resolution of measurements and the time lag between the discharge or precipitation events and subsequent RWQE. A decision model was successful in predicting RWQEs using either large discharge or precipitation events at most stations within the mouth of the KA system. Precipitation events were slightly better indicators (high precipitation events preceded RWQEs at 28 out of 30 stations as compared to 25 out of 30 for discharge events). The difference was more pronounced for the more developed Saco River watershed. The decision model using river discharge failed to predict most RWQEs in the Saco River watershed (high discharge events preceded RWQEs at only eight of 31 stations). Precipitation was a more effective indicator (high precipitation events occurred within 3 days of RWQEs at 23 of 31 stations), but still less accurate than in the KA system. Calibration of the model using precipitation at different locations (i.e. TRMM squares) enables the model to discriminate between the effects of run-off at different locations (as opposed to discharge which integrates run-off over all areas of the watershed) and allowed for greater accuracy than the discharge decision model.

The limited ability of a decision model using only one parameter to predict RWQEs is not unprecedented. Sampson *et al*. [40] found no significant relationship between precipitation and water quality at Lake Superior beaches. The lack of a simple relationship motivated us to examine additional mechanisms when analysing the higher frequency sampling of study II. *Escherichia coli* (figure 8) and total coliform (figure 9) concentrations collected at the five locations within Saco Bay from 2010 to 2012 during study II were highly variable.

The pronounced along-river and along-coast gradient in both *E. coli* and total coliform concentrations is consistent with the main source of faecal pollution originating upstream in the Saco River from the four WWTPs and other sources of pollution. However, there are most likely additional sources outside of the river. Faecal pollution from beach goers and their pets is a possible source of *E. coli* and total coliforms in coastal waters along the beach [58,59]. The lack of correlation between *E. coli* concentrations at the stations within the river and those along the coast as well as the additional peak in *E. coli* concentrations in the summer at the stations along the coast (when beaches in Maine are most populated) is consistent with a second source of faecal pollution on the coast. Total coliform concentrations show the same pattern. However, the reduction in concentrations of both *E. coli* and total coliforms as one moves north away from the Saco River mouth suggests that this is a secondary source.

The strong seasonal signals of the *E. coli* and total coliform concentrations within the Saco River and near the mouth of the Scarborough River are consistent with temporal variations in the population of the area. The lag times for the seasonal signal (equation (2.1)) range from 171 to 269 days (tables 4 and 5), resulting in peak values for the seasonal signal that range from 21 June to 27 September, which encompass the summer season when the population of southern Maine increases owing to an influx of tourists (and the associated sewage outflow is greatest).

There are different mechanisms responsible for variation in water quality at different locations within the Saco Bay region. The negative correlations of *E. coli* concentrations with river discharge at the two stations within the river and total coliform concentrations at the Saco station suggest that river discharge is not simply a supply mechanism for faecal pollution that transports run-off and sewage downstream to the lower portions of the river. While faecal pollution does travel down the river to the open ocean, high values of river discharge increase the amount of water in the Saco River and tend to dilute the concentrations of *E. coli* and total coliforms. This finding is consistent with the decision model that found discharge was a poor predictor of RWQEs in the Saco River and adjacent coastal ocean. Precipitation is positively correlated with *E. coli* concentrations at the Camp Ellis and coastal stations and with total coliforms at both the Saco and Scarborough stations, indicating that precipitation (and the subsequent run-off) is a slightly better predictor than discharge of reduced water quality.

The locations within the watershed where precipitation values were correlated with water quality at the stations in study I were generally higher in urban development or agriculture and/or closer to the water quality stations (tables 4 and 5, and figure 1). Both *E. coli* and total coliform concentrations at the Scarborough station and total coliform concentration at the Saco station were most correlated with precipitation with TRMM square 3, which encompasses or is adjacent to these two stations. It is also home to the greatest development in the watershed. Interestingly, concentrations at the Scarborough station lag precipitation by 3 days, suggesting that run-off is transported by the Saco River and then the river plume to the Scarborough station. *Escherichia coli* concentrations at the Camp Ellis station were most correlated with precipitation at TRMM square 92, which is far up the estuary (and consistent with a 2-day lag) but contains the second most urban development in the watershed. However, the link between land use, precipitation and water quality is not straightforward for all stations. *Escherichia coli* concentrations at the Old Orchard Beach station were also correlated with precipitation at TRMM square 10, which contain little development. The mechanism that links the precipitation at these locations to reduced water quality warrants further study (e.g. [11]).

The wind-driven advection of the plume emanating from the Saco River affects water quality along the coast of Saco Bay. Fortier [57] showed that easterly or northeasterly winds result in onshore movement of the Saco river plume, while westerly or southwesterly winds result in offshore movement (figure 3*b*). The correlation between winds from the northeast and *E. coli* concentrations at the Camp Ellis and coastal stations (as well as the significant correlation between easterly/northeasterly winds and total coliform concentrations at all but one station) is consistent with wind-driven onshore movement of the Saco River plume (carrying high *E. coli* and total coliform concentrations) affecting water quality along the beach. The relationship between winds and water quality has been confirmed by earlier studies. Nevers & Whitman [3] found that their model performance was strongly affected by wind direction, while Olyphant & Whitman [42] found that onshore winds were significantly correlated with *E. coli* concentrations in Lake Michigan.

The increase in correlation of the multiple linear regression models at other stations when including concentrations from the Saco station is consistent with the Saco River acting as a source for both *E. coli* and total coliforms. Thupaki *et al.* [5] used a budget analysis of *E. coli* in Lake Michigan to examine the effects of river plumes on water quality in the region and found similar results. Nevers & Whitman [3] showed that the incorporation of *E. coli* concentrations from small outfalls that emptied near their sites resulted in marked gains in model outcome. Although not specifically tested in this study, the higher *E. coli* and total coliform concentrations within the river suggest that the river plume should also contain high *E. coli* and total coliform concentrations that would result in reduction of coastal water quality wherever the plume encounters the coast.

The correlation values for the multiple linear regression models of *E. coli* and total coliforms are similar to previous studies. Several studies of beaches on Lake Michigan [3,20,42] found correlation values between their various models and *E. coli* concentrations that ranged from 0.56 to 0.84. The reliance of the multiple linear regression models in this study on different parameters from models at other locations is consistent with earlier studies (e.g. [56]), which found that general models were not able to predict water quality at multiple sites and instead, the models must be built and calibrated for individual sites.

In conclusion, an examination of physical mechanisms on two different watersheds, their rivers and the coastal ocean adjacent to their river mouths in the Gulf of Maine revealed that physical mechanisms have a significant effect on river and coastal water quality. The varying effects of these different mechanisms at different locations indicate that successful prediction of water quality requires an understanding of multiple inputs such as land use, river discharge, precipitation, seasonality and coastal circulation. The inclusion of hydrometeorological processes such as salinity, winds and seasonality helps in the accurate prediction of water quality in Saco Bay and shows that coastal oceanic circulation (primarily through transport of the river plume) both contributes to the transport of faecal pollution and has a direct effect on water quality in this region. We found that multiple linear regression models that incorporate only easily measured hydrometeorological parameters are able to successfully predict a significant amount of the variation in *E. coli*and total coliform concentrations at all stations within Saco Bay. Although the inclusion of total coliform and *E. coli* concentrations from upstream increased the ability of the model to explain the variance of downstream stations, it would not improve the immediate use of this particular model as acquiring this information still requires culturing bacteria. However, because the ultimate source of faecal contamination at Saco is largely point-source discharge from combined sewer overflows, empirical measurement of indicator bacteria at this location might be replaced with another regression model that predicted discharges as a function of recent and historical precipitation. A number of studies have examined the relationship between water quality and hydrometeorological parameters at freshwater beaches. To our knowledge, this is one of the first attempts to create a mechanistic model to predict water quality in marine waters and is the first attempt to create a model for coastal Maine. The systemic lags of the hydrometeorological processes in the multiple linear regression models provide the ability to predict high *E. coli* and total coliform concentrations and allow coastal managers to concentrate their limited resources at the appropriate times to test regions for faecal pollution. While the models are not able to capture all of the variation of water quality at the five stations in Saco Bay, their performance is similar to models in other regions, which have been incorporated into water resource managers' decisions, indicating that the use of a mechanistic model in coastal Maine is feasible.

## Supplementary Material

saco_rsos.csv - comma separated variable file that contains date, time, E. coli concentrations, total coliform concentrations, and salinity at the Saco station.

## Supplementary Material

une_rsos.csv - comma separated variable file that contains date, time, E. coli concentrations, total coliform concentrations, and salinity at the UNE station.

## Supplementary Material

campellis_rsos.csv - comma separated variable file that contains date, time, E. coli concentrations, total coliform concentrations, and salinity at the Camp Ellis station.

## Supplementary Material

scar_rsos.csv - comma separated variable file that contains date, time, E. coli concentrations, total coliform concentrations, and salinity at the Scarborough station.

## Supplementary Material

oob_rsos.csv - comma separated variable file that contains date, time, E. coli concentrations, total coliform concentrations, and salinity at the Old Orchard Beach station.

## Data accessibility

All water quality and hydrometeorological data are included as the electronic supplementary material.
